# Supplementary figures and images for: Movement History Influences Pendulum Test Kinematics in Children With Spastic Cerebral Palsy
Source: Front Bioeng Biotechnol. 2020 Aug 7;8:920. doi: 10.3389/fbioe.2020.00920 (PMC7426371; doi:10.3389/fbioe.2020.00920)

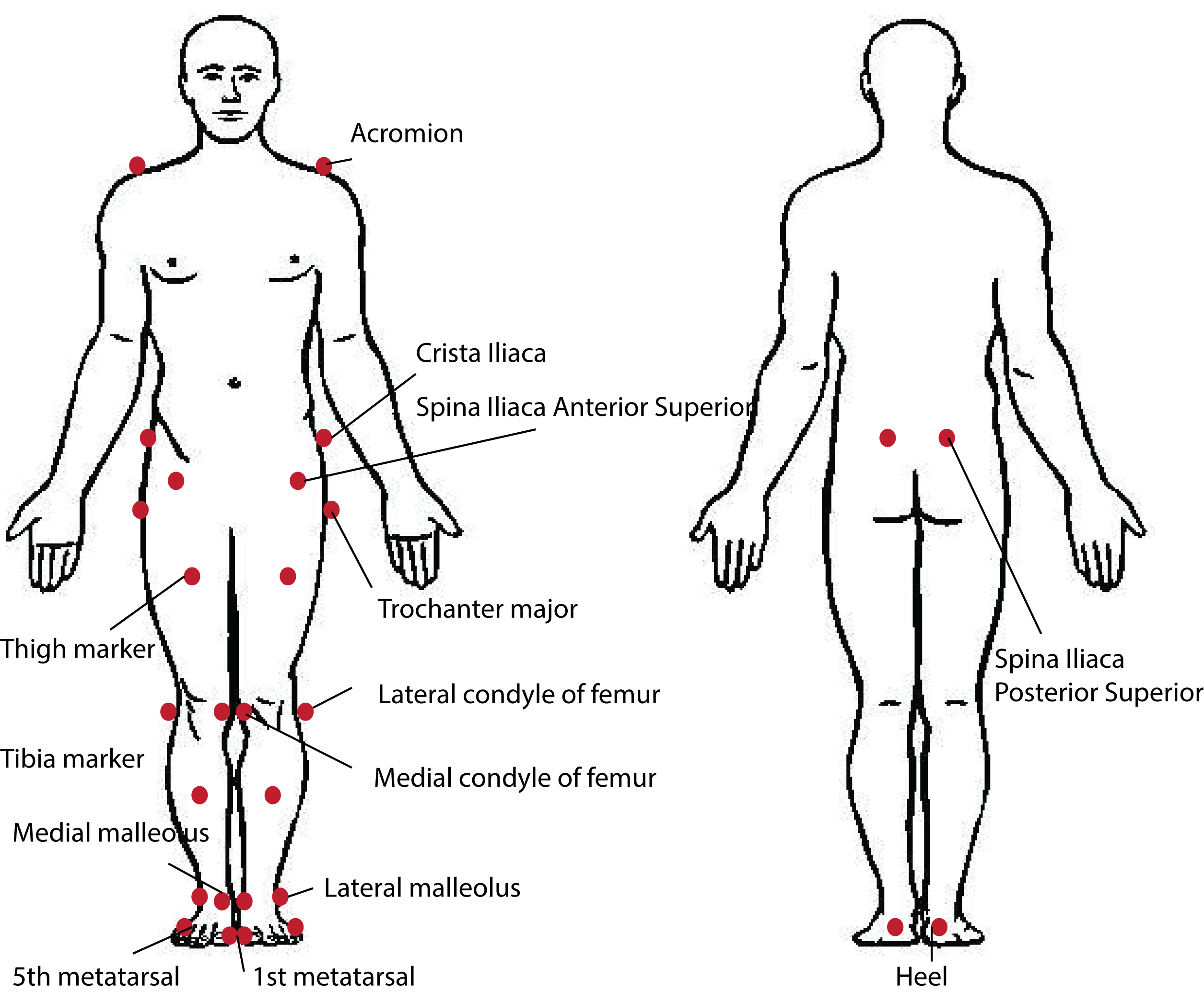

Supplement: FIGURE S1 — Marker set-up used to measure kinematics during the pendulum test in children with CP and TD children. [file Image_1.TIF]

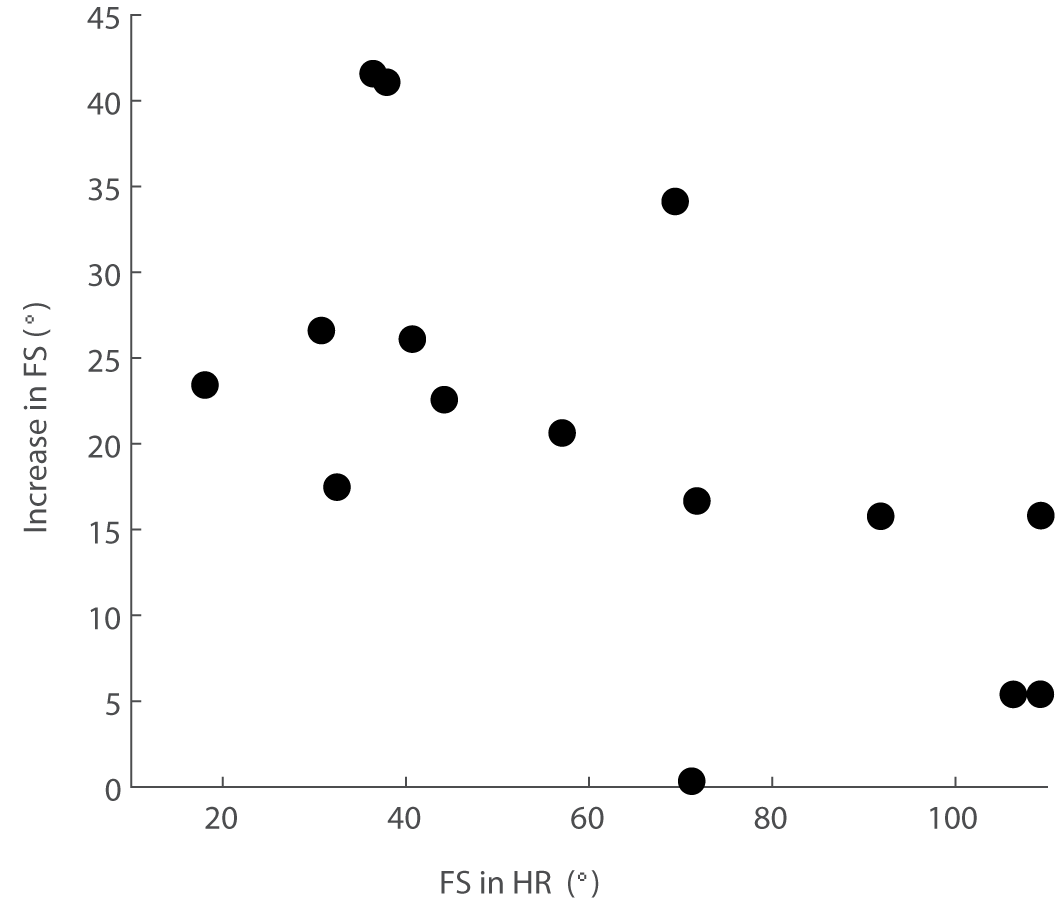

Supplement: FIGURE S2 — Relation between first swing excursion in the isometric condition and the increase in first swing excursion in the pre-movement with respect to the isometric condition for children with CP in the sitting position. [file Image_2.TIF]

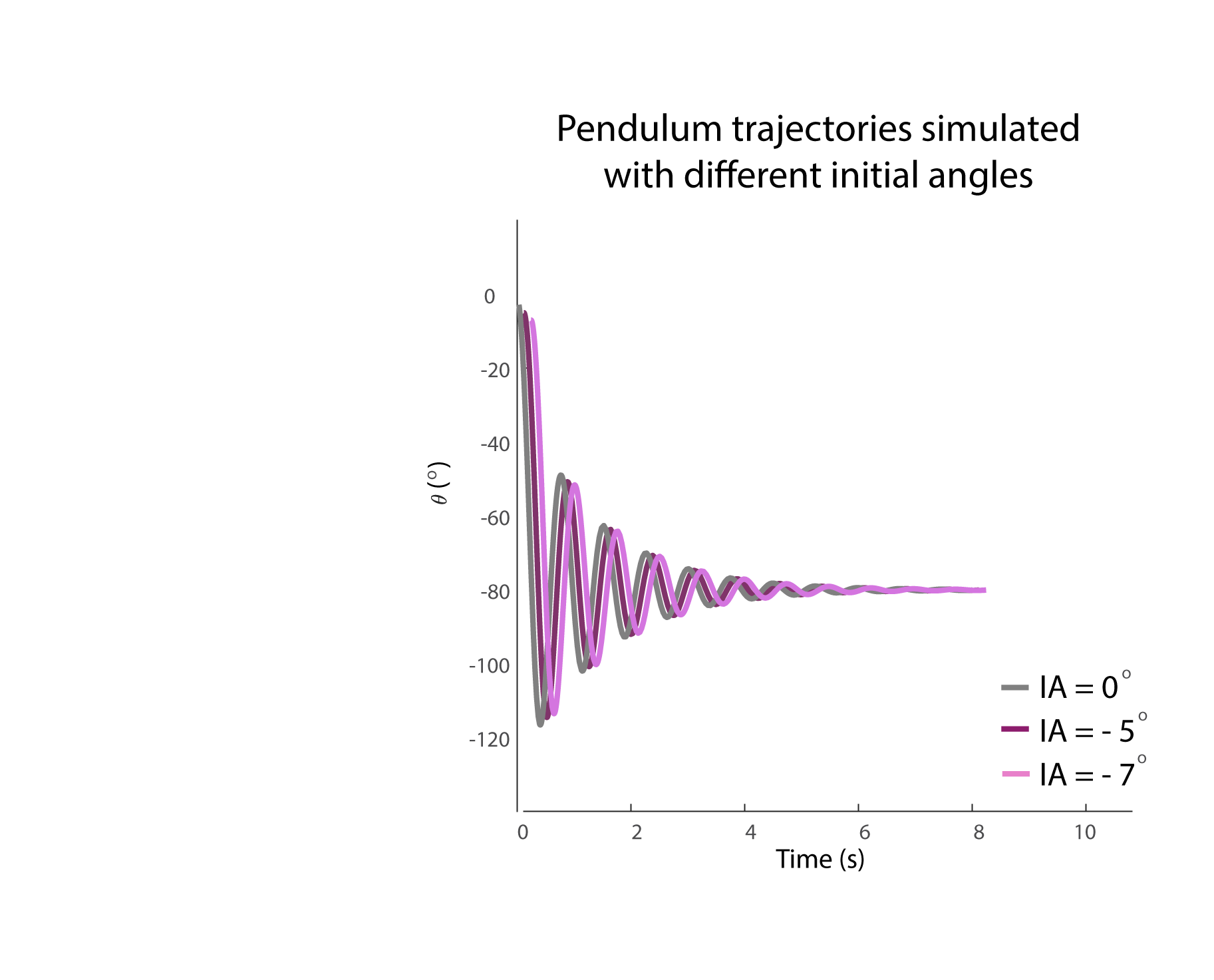

Supplement: FIGURE S3 — Simulated pendulum movement for three different initial angles: 0° (gray), –5° (purple), –7° (pink). Only minor differences are observed in the first swing excursion between trials. Curves are shifted horizontally for clarity. Kinematic trajectories were simulated based on the model described by De Groote et al. (2018). The model with short-range stiffness, but without reflex activity was used, since the largest differences based on initial angle were expected in this configuration. Muscle tone was set on 0.4 Nm. We used values for mass (2.4 kg), length (0.26 m), and inertia (0.21 kg/m2) that were representative for the subjects in our database. [file Image_3.tif]
