# Supplementary material for: Movement History Influences Pendulum Test Kinematics in Children With Spastic Cerebral Palsy
Source: Front Bioeng Biotechnol. 2020 Aug 7;8:920. doi: 10.3389/fbioe.2020.00920 (PMC7426371; doi:10.3389/fbioe.2020.00920)
Supplement: TABLE S1 — Spasticity scores as measured by the Modified Ashworth Scale for children with CP. [file Table_1.docx]

# Table S1: Spasticity scores as measured by the Modified Ashworth Scale for children with CP

|  | **GMFCS** | **Quadriceps** | |  | **Hamstrings** | | **Measured leg** |
| --- | --- | --- | --- | --- | --- | --- | --- |
|  | **level** | *L* | *R* |  | *L* | *R* |  |
| **S1** | 1 | 0 | 0 |  | 1 | 0 | L |
| **S2** | 1 | 0 | 0 |  | 0 | 0 | L |
| **S3** | 1 | 0 | 0 |  | 1.5 | 0 | L |
| **S4** | 2 | 1.5 | 1.5 |  | 1.5 | 2 | R |
| **S5** | 2 | 0 | 0 |  | 1 | 0 | L |
| **S6** | 1 | 0 | 0 |  | 1 | 1.5 | R |
| **S7** | 1 | 0 | 0 |  | 0 | 0 | R |
| **S8** | 1 | 2 | 0 |  | 1.5 | 0 | R |
| **S9** | 2 | 0 | 0 |  | 1.5 | 1.5 | R |
| **S10** | 2 | 0 | 2 |  | 1 | 1 | R |
| **S11** | 3 | 0 | 1 |  | 2 | 2 | R |
| **S12** | 3 | 0 | 0 |  | 1.5 | 1 | R |
| **S13** | 1 | 0 | 0 |  | 0 | 1 | R |
| **S14** | 2 | 0 | 0 |  | 1.5 | 3 | R |
| **S15** | 1 | 0 | 0 |  | 1 | 1 | R |
