# Supplementary material for: Movement History Influences Pendulum Test Kinematics in Children With Spastic Cerebral Palsy
Source: Front Bioeng Biotechnol. 2020 Aug 7;8:920. doi: 10.3389/fbioe.2020.00920 (PMC7426371; doi:10.3389/fbioe.2020.00920)
Supplement: TABLE S2 — (a) Key kinematic outcomes (mean and standard deviation). (b) p-values for the comparisons between subject groups (CP and TD) and positions (sitting and supine). [file Table_2.docx]

# Table S2: a) Key kinematic outcomes (mean and standard deviation). b) p- values for the comparisons between subject groups (CP and TD) and positions (sitting and supine).

| a) | **Sit (HR)** | | | | **Supine (HR)** | | | |
| --- | --- | --- | --- | --- | --- | --- | --- | --- |
|  | **CP** | | **TD** | | **CP** | | **TD** | |
|  | *Mean* | *SD* | *Mean* | *SD* | *Mean* | *SD* | *Mean* | *SD* |
| **FS (°)** | 62 | 30 | 113 | 8 | 61 | 29 | 105 | 8 |
| **NO (#)** | 3.7 | 2.0 | 6.4 | 1.4 | 4.2 | 1.8 | 5.7 | 1.6 |
| **RA (°)** | 64 | 7 | 66 | 7 | 53 | 9 | 59 | 6 |

| b) | **CP vs. TD** | | **Sit vs. Supine** | |
| --- | --- | --- | --- | --- |
|  | **Sit** | **Supine** | **CP** | **TD** |
| **FS** | < 0.001 | < 0.001 | 0.90 | <0.001 |
| **NO** | < 0.001 | < 0.05 | 0.06 | 0.07 |
| **RA** | 0.32 | < 0.05 | <0.001 | <0.001 |
