# Supplementary material for: Movement History Influences Pendulum Test Kinematics in Children With Spastic Cerebral Palsy
Source: Front Bioeng Biotechnol. 2020 Aug 7;8:920. doi: 10.3389/fbioe.2020.00920 (PMC7426371; doi:10.3389/fbioe.2020.00920)
Supplement: TABLE S3 — (a) Influence of movement history on key kinematic outcomes (mean and standard deviation). (b) p-values for the comparison between conditions (isometric vs. pre-movement). (c) p-values for the comparison of the influence of pre-movement on the key kinematic outcomes between groups (CP vs TD) for the outcomes that were influenced by pre-movement. [file Table_3.docx]

# Table S3: a) Influence of movement history on key kinematic outcomes (mean and standard deviation). b) p-values for the comparison between conditions (isometric vs. pre-movement). c) p-values for the comparison of the influence of pre-movement on the key kinematic outcomes between groups (CP vs TD) for the outcomes that were influenced by pre-movement.

| a) | **Sit** | | | | **Supine** | | | |
| --- | --- | --- | --- | --- | --- | --- | --- | --- |
|  | **CP** | | **TD** | | **CP** | | **TD** | |
|  | *Mean* | *SD* | *Mean* | *SD* | *Mean* | *SD* | *Mean* | *SD* |
| **Fs (°)** | 21 | 12 | 8 | 7 | 10 | 7 | 9 | 5 |
| **No (#)** | 1.7 | 1.1 | 0.4 | 0.4 | 0.7 | 0.8 | 0.5 | 0.7 |
| **Ra (°)** | 64 | 6 | 66 | 7 | 3 | 8 | 59 | 6 |

| b) | **Sit** | | **Supine** | |
| --- | --- | --- | --- | --- |
|  | **CP** | **TD** | **CP** | **TD** |
| **Fs** | < 0.001 | < 0.001 | < 0.001 | < 0.001 |
| **No** | <0.001 | < 0.01 | < 0.01 | < 0.05 |
| **Ra** | 0.98 | 0.26 | 0.11 | 0.27 |

| c) | **CP vs TD** | | **Sit vs. Supine** | |
| --- | --- | --- | --- | --- |
|  | **Sit** | **Supine** | **CP** | **TD** |
| **Fs** | < 0.005 | 0.47 | < 0.01 | 0.66 |
| **No** | < 0.001 | 0.45 | < 0.01 | 0.68 |
