# Supplementary material for: Movement History Influences Pendulum Test Kinematics in Children With Spastic Cerebral Palsy
Source: Front Bioeng Biotechnol. 2020 Aug 7;8:920. doi: 10.3389/fbioe.2020.00920 (PMC7426371; doi:10.3389/fbioe.2020.00920)
Supplement: TABLE S4 — (a) EMG-based outcomes describing rectus femoris reflex activity (occurrence of reflex activity (%), onset of reflexes (ms) AUC, mean and standard deviation). (b) p-values for the comparisons between subject groups (CP and TD) and positions (sitting and supine). [file Table_4.docx]

Table S4: a) EMG-based outcomes describing rectus femoris reflex activity (occurrence of reflex activity (%), onset of reflexes (ms) AUC, mean and standard deviation). b) p- values for the comparisons between subject groups (CP and TD) and positions (sitting and supine).

| a) | **Sit (HR)** | | | | **Supine (HR)** | | | |
| --- | --- | --- | --- | --- | --- | --- | --- | --- |
|  | **CP** | | **TD** | | **CP** | | **TD** | |
|  | *Mean* | *SD* | *Mean* | *SD* | *Mean* | *SD* | *Mean* | *SD* |
| **Occurrence (%)** | 74 | 40 | 42 | 38 | 63 | 41 | 29 | 27 |
| **Reflex onset (ms)** | 142 | 30 | 212 | 78 | 165 | 77 | 294 | 124 |
| **AUC** | 0.0044 | 0.0029 | 0.0013 | 0.0010 | 0.0035 | 0.0021 | 0.0012 | 0.0009 |

| b) | **CP vs. TD** | | **Sit vs. Supine** | |  |
| --- | --- | --- | --- | --- | --- |
|  | **Sit** | **Supine** | **CP** | **TD** | |
| **Occurrence (%)** | < 0.05 | < 0.05 | 0.11 | 0.16 | |
| **Reflex onset (ms)** | < 0.05 | < 0.001 | 0.56 | 0.05 | |
| **AUC** | < 0.001 | < 0.001 | 0.17 | 0.17 | |
