# Supplementary material for: Movement History Influences Pendulum Test Kinematics in Children With Spastic Cerebral Palsy
Source: Front Bioeng Biotechnol. 2020 Aug 7;8:920. doi: 10.3389/fbioe.2020.00920 (PMC7426371; doi:10.3389/fbioe.2020.00920)
Supplement: TABLE S5 — (a) Influence of movement history on EMG-based outcomes describing rectus femoris reflex activity (mean and standard deviations). (b) p-values for the comparison between conditions (isometric and pre-movement). (c) p-values for the comparison between groups for parameters that were influenced by pre-movement. [file Table_5.docx]

Table S5: a) Influence of movement history on EMG-based outcomes describing rectus femoris reflex activity (mean and standard deviations). b) p-values for the comparison between conditions (isometric and pre-movement). c) p-values for the comparison between groups for parameters that were influenced by pre-movement.

| a) | **Sit** | | | | **Supine** | | | |
| --- | --- | --- | --- | --- | --- | --- | --- | --- |
|  | **CP** | | **TD** | | **CP** | | **TD** | |
|  | *Mean* | *SD* | *Mean* | *SD* | *Mean* | *SD* | *Mean* | *SD* |
| **Trials (%)** | 13 | 24 | 5 | 32 | 3 | 22 | 5 | 26 |
| **Timing (ms)** | 87 | 100 | 61 | 89 | 78 | 36 | -19 | 108 |
| **AUC** | 0.12 | 0.33 | 0.001 | 0.1195 | -0.03 | 0.20 | -0.02 | 0.13 |

| b) | **Sit** | | **Supine** | |
| --- | --- | --- | --- | --- |
|  | **CP** | **TD** | **CP** | **TD** |
| **Trials** | 0.07 | 0.55 | 0.64 | 0.53 |
| **Timing** | < 0.05 | 0.08 | < 0.001 | 0.68 |
| **AUC** | <0.05 | 0.98 | 0.58 | 0.49 |

| c) | **CP vs TD** | | **Sit vs. Supine** | |
| --- | --- | --- | --- | --- |
|  | **Sit** | **Supine** | **CP** | **TD** |
| **Timing** | 0.58 | < 0.05 | 0.78 | 0.22 |
