# Supplementary material for: Movement History Influences Pendulum Test Kinematics in Children With Spastic Cerebral Palsy
Source: Front Bioeng Biotechnol. 2020 Aug 7;8:920. doi: 10.3389/fbioe.2020.00920 (PMC7426371; doi:10.3389/fbioe.2020.00920)
Supplement: TABLE S6 — (a) Correlations between first swing excursion and resting angle in the isometric (HR) condition. (b) Correlations between first swing excursion and occurrence of reflexes in the isometric (HR) condition. [file Table_6.docx]

Table S6: a) Correlations between first swing excursion and resting angle in the isometric (HR) condition. b) Correlations between first swing excursion and occurrence of reflexes in the isometric (HR) condition.

| a) | **CP** | | **TD** | |
| --- | --- | --- | --- | --- |
|  | *r* | *p* | *r* | *p* |
| **Sit** | 0.29 | 0.30 | 0.01 | 0.97 |
| **Supine** | 0.84 | <0.001 | 0.66 | < 0.01 |

| b) | **CP** | | **TD** | |
| --- | --- | --- | --- | --- |
|  | *r* | *p* | *r* | *p* |
| **Sit** | - 0.82 | < 0.01 | - 0.34 | 0.21 |
| **Supine** | - 0.87 | < 0.01 | - 0.05 | 0.86 |
